# Factors affecting the outcome of full pulpotomy in permanent posterior teeth diagnosed with reversible or irreversible pulpitis

**DOI:** 10.1038/s41598-022-24815-0

**Published:** 2022-11-24

**Authors:** Min Zhang, Yuhua Xiong, Xuerong Wang, Yunqin Wang, Yixin Cai, Junchen Xu, Chengfei Zhang, Jin Li

**Affiliations:** 1 Department of Geriatric Dentistry, The Affiliated Stomatological Hospital of Nanjing Medical University, 136 Hanzhong Road, Nanjing, 210029 People’s Republic of China; 2 Jiangsu Province Key Laboratory of Oral Diseases, Nanjing, People’s Republic of China; 3 Jiangsu Province Engineering Research Center of Stomatological Translational Medicine, Nanjing, People’s Republic of China; 4 Restorative Dental Sciences, Faculty of Dentistry, The University of Hong Kong, Pok Fu Lam, Hong Kong, Special Administrative Region People’s Republic of China

**Keywords:** Diseases, Health care, Medical research

## Abstract

This study aimed to investigate the factors affecting the success rate of full pulpotomy in permanent posterior teeth with pulpitis. The study included 105 permanent posterior teeth clinically diagnosed as reversible or irreversible pulpitis in 92 patients aged 18–82 years. All teeth underwent a full pulpotomy using mineral trioxide aggregate as a capping material and were recalled for clinical and radiographic evaluation at 3, 6, 12, and 24 months postoperatively. The overall success rate after the 12-month review was above 90%, and failed cases mainly occurred during the first 12 months after treatment. In this study, the treatment outcome of pulpotomy was not related to sex, or tooth position and the cause of pulpitis. To analyze the influence of age on the treatment outcome, all the teeth were allocated to three groups: group 1 (18–39 years); group 2 (40–59 years); and group 3 (≥ 60 years). A significant difference in success rate was found between groups 1 and 3 (P = 0.014). These results suggest that pulpotomy can be used as an alternative treatment for permanent mature teeth diagnosed with pulpitis and that aging is one factor affecting the treatment outcome.

## Introduction

The dentin/pulp complex undergoes physiological changes throughout its life under normal circumstances, as well as providing pathological defensive responses to injuries, caries, and operative procedures, resulting in gradual narrowing of the pulp volume and circumference^1^. The dentin/pulp complex plays an essential role in maintaining tooth integrity and function by supporting it with nutrition and protecting it against microbial invasion^2,3^. Aging not only affects the structure and anatomy of the pulp chamber but also reduces the capacity of pulp tissue to maintain physiological homeostasis and the healing process^4,5^. In addition to continuous dentin apposition, age-related pulp transitions include fibrosis, degeneration of odontoblasts, and reduced cell density^5,6^. A histological study reported that the teeth of older subjects have lower numbers of odontoblasts and progenitor cells^7^. Furthermore, the biological function, proliferation, and differentiation of cells were more profound in young pulps compared with older pulps, and the apoptosis pathway was also more highly expressed in older pulps^8^. All of these alterations could affect the reparative potential of the pulp, rendering the aged pulp tissue vulnerable to external insults^9,10^.

Pulpotomy, which has been conventionally used to treat young permanent teeth to promote their root development, has been recently applied to mature permanent teeth diagnosed with irreversible pulpitis in clinical studies^11–15^. With the improvement of pulp capping materials, the success rate of pulpotomy in human mature permanent teeth seems promising^16,17^. However, due to a lack of well-established correlations between clinical symptoms and histologic findings, the inflammatory state of the pulp is determined by clinical examination, such as pain intensity and duration, and clinical observation under a microscope, such as the color of blood and the bleeding time, which could be incorrect^18^, leaving infected pulp unremoved. In such a scenario, the pulpotomy would fail.

Previous clinical studies on pulpotomy in mature permanent teeth have included patients aged from 18 to 68 years, although age has not been considered a factor in the treatment outcome^12,13^. Nevertheless, a recent study found a significant difference between adult patients aged under 40 years and those aged above 40 years in the success rate of direct pulp capping^19^. With limited studies in the aging population, the feasibility and success rate of pulpotomy in this population warrant further investigation.

This study aimed to evaluate the clinical outcomes of full pulpotomy in patients of different ages with pulpitis and to explore the potential factors that influence the outcome of clinical treatment, to provide more evidence for clinical application.

## Material and methods

### Ethics

All subjects gave their informed consent for inclusion before they participated in the study. The study was conducted in accordance with the Declaration of Helsinki, and the protocol was approved by the Ethics Committee of the Institutional Ethics Committee at Nanjing Medical University (PJ2018-058-001). This report is part of a larger study, which has been registered at the Chinese Clinical Trial Registry (Identifier: ChiCTR2200055904).

### Sample size calculation

The sample size calculation for the outcome assessment of pulpotomy was based on the results of Taha and Khazali (2017) that the success rate of MTA pulpotomy in mature permanent teeth with irreversible pulpitis was 83%^20^. Considering the anticipated success in the present study group as 93%, and for the study with 80% power and a = 0.05, the minimum sample size was calculated to be 90 teeth. Anticipating about 15% loss during the follow-ups, it was planned to enroll 105 teeth in the study.

### Inclusion/exclusion criteria

Selection and recruitment extended from May 2019 to April 2021, and included a total of 124 teeth. Five teeth were excluded during the preoperative evaluation, and 14 were excluded during the pulpotomy procedure. A total of 105 teeth from 92 patients were clinically diagnosed with reversible pulpitis or irreversible pulpitis and were included according to the following criteria (Tables 1 and 2).

**Table 1:** Inclusion criteria.

**Inclusion criteria**
The patients are over 18 years old and have no history of systemic disease that affects oral treatment
Mature permanent molar teeth with pulp injury, involved both reversible pulpitis or irreversible pulpitis, due to caries or abrasion
Discomfort triggered by cold/sweet stimuli, which relieves within 30 s after the removal of the stimulus. Or discomfort/pain triggered by a cold/sweet stimulus, which lasts more than 30 s, but no more than 10 min after the stimulus removal. The pain is non-spontaneous. No history of night pain; No discomfort in percussion
The remaining tooth tissue has enough space for restoration
Having healthy periodontal tissue
Radiograph showed no obvious abnormality around the apex of the tooth, PAI ≤ 1
After excavation, red homogeneous and blood-filled tissue was observed on the surface of the pulp wound; hemostasis could be achieved within 5 min

**Table 2:** Exclusion criteria.

**Exclusion criteria**
Primary teeth or permanent teeth with immature roots
It is caused by the wedge-shaped defect, cracked tooth, or tooth fracture. Or after meticulous caries excavation, no exposures appear
Sensitive to heat, persistent irritating pain or dull throbbing pain, sharp spontaneous pain, and tenderness to percussion or pain exacerbated by lying down
The remaining tooth tissue has no restorative value
Periodontal tissue is unhealthy
PAI ≥ 2
After excavation, tissue was observed unhealthy on the surface of the pulp wound and could not achieve hemostasis within 5 min

### Clinical procedure

The preoperative and postoperative periapical radiographs were taken using film holders (Acteon, Mérignac, France) with a parallel technique, and assessed by two experienced endodontists. Both observers were experienced endodontists and were blinded to the treatment procedures used. They were calibrated by diagnosing radiographs. The intraexaminer reliability for the periapical index (PAI) calibration results was κ = 0.84, indicating ‘‘almost perfect agreement’’; the interexaminer agreement (examiner scores vs the calibration kit ‘‘authorized scores’’) was κ = 0.80, indicating ‘‘substantial agreement’’**.** The periapical index (PAI) was used to evaluate the apical condition during diagnosis and at the follow-up visits as described previously^21^. All teeth underwent a complete pulpotomy using mineral trioxide aggregate as a capping material and were recalled for clinical and radiographic evaluation at 3, 6, 12, and 24 months postoperatively.

Teeth with severe tooth wear diagnosed as reversible pulpitis were treated with desensitizer (Gluma, Kulzer, Germany) twice in two separate weekly visits before including. If the symptoms were not improved after the desensitization therapy, pulpotomy was conducted.

Carious symptomatic teeth that were included were verified carefully according to the inclusion criteria. In addition to the preoperative examination, a rigorous evaluation during the procedure was conducted. On the basis of direct observation of dentin and the exposed pulp tissue under deep caries^22^, teeth that only required cavity restoration, pulp capping, or pulpectomy were excluded. Teeth with sound dentin at the bottom of the cavity or around the exposure, those with continuous blood-filled pulp and no dentin chips displaced, and those with no continuous pulp tissue and bleeding that could not be controlled within 5 min were excluded.

The clinical treatment and the follow-up clinical examination were conducted by one endodontic postgraduate student under the supervision of an endodontic professor. The procedure was as follows: patients were asked to rinse their mouth with 3% H_2_O_2_ for 30 s after local infiltration with 1.7 mL 4% Articaine with 1:100,000 epinephrine (Septodont, Saint-Maur-Desfosses, France). A rubber dam was correctly applied and disinfected with a 75% alcohol pellet before caries excavation. Dental caries were thoroughly removed with a low-speed round bur under an endodontic microscope. Then, the coronal pulp was amputated with a 1 mm diameter curette (exc246, HuFriedy, Chicago, IL, USA). Hemostasis was achieved within 5 min after rinsing with 0.9% saline. A 2-mm-thick layer of mineral trioxide aggregate (MTA) (ProRoot MTA, Dentsply, Tulsa, OK, USA) was mixed and placed on top of the remaining root pulp with gentle pressure with a clean cotton pellet. After the initial set, a layer of flowable composite (Filtek Supreme, 3 M, St. Paul, USA) was placed over the MTA and light cured. Glass ionomer cement was placed as a temporary restoration and then replaced with resin composite (Z250, 3 M, ESPE), or a computer-aided design/manufacture (CAD/CAM) ceramic onlay (3shape, Copenhagen K, Denmark), based on the patient’s choice at the second visit 2 weeks later. Postoperative pain intensity was recorded at the second visit. Postoperative pain was recorded on a 0 to 10-cm visual analog scale (VAS) scale every 24 h until the second visit (14 days) by patients. The reports were submitted to the two endodontists for evaluation.

### Evaluation of the outcome

Recall visits were arranged at 3, 6, 12, and 24 months postoperatively, and both clinical and radiographic examinations were conducted by the same postgraduate student under supervision. The discoloration of the treated teeth was recorded as “Yes/No” by observing the color difference between the treated teeth and the other healthy teeth next to them.

The success of the treatment was evaluated by combining the patient’s subjective feelings with the results of the clinical examination and the periapical radiograph^23^. The treatment was considered successful if there was (1) absence of pain or discomfort from the tooth; (2) absence of discomfort during the clinical examination including probing, percussion, and cold testing; and (3) absence of radiographic signs of root resorption, calcification, fracture, furcal rarefaction, or apical periodontitis.

## Results

**Figure 1:**
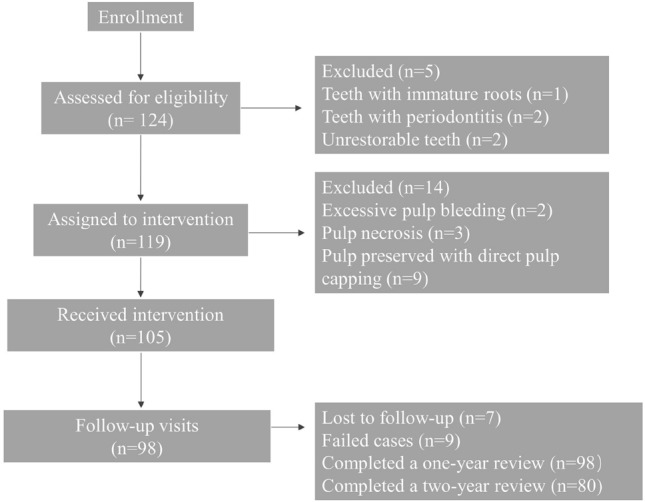
Flow chart of the current progress of this ongoing clinical study.

The procedure of the study is presented in a flow chart (Fig. 1). In total, 105 molar teeth in 92 patients aged 18–82 years (median age 43 years) underwent a full pulpotomy. 98 of them presented the 12-month follow-up. Of the 7 cases lost to follow-up, three were absent from the 3-month review, three were from 6 months, and the other one was from the 12-month review. The overall success rate was 90.8% at the 12-month review. The average treatment duration was 48.7 min. Over half of the patients (54.3%) reported no pain after the anesthesia wore off.

**Figure 2:**
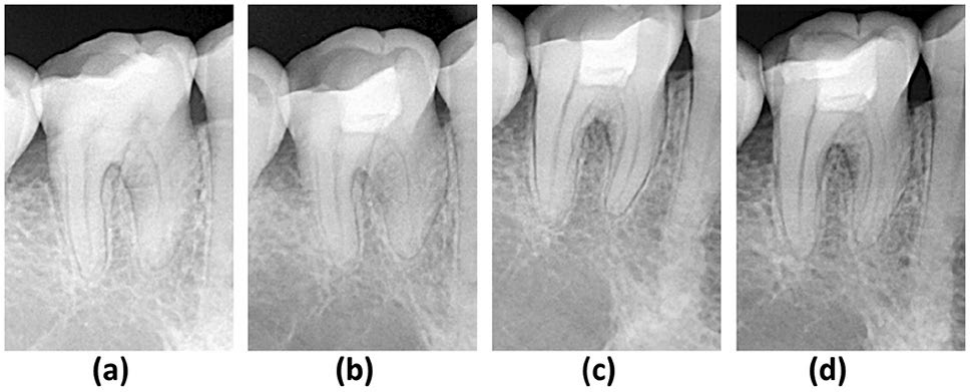
Radiographs of a right mandibular first molar with severe tooth wear in a 62-year-old woman. (**a**) Preoperative radiograph showing the reduced size of the pulp chamber. (**b**) 3-month follow-up. (**c**) 6-month follow-up. (**d**) 12-month follow-up.

**Figure 3:**
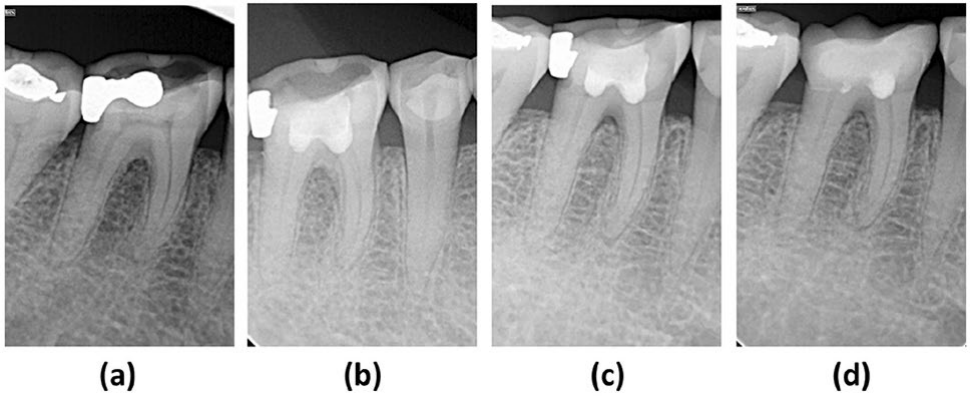
Radiographs of a right mandibular first molar with severe tooth wear and secondary caries in a 63-year-old woman. (**a**) Preoperative radiograph showing occlusal abrasion and a disto-occlusal amalgam restoration with secondary caries. (**b**) 3-month follow-up. (**c**) 6-month follow-up. (**d**) A ceramic inlay restoration was completed at the 12-month follow-up.

Table 3 shows the follow-up examination outcomes of 98 teeth at 12 months postoperatively. During the follow-up, age was found to be one of the factors influencing the outcome. Therefore, all the included teeth were allocated into 3 groups to analyze the impact of age on the treatment outcomes then. The overall success rates for each age group were 100%, 85.7%, and 81.5% for groups 1, 2, and 3 respectively. Significant differences in success rate were found between groups 1 and 2 (P = 0.043), and groups 1 and 3 (P = 0.014), whereas no significant differences were observed between groups 2 and 3 (P ≥ 0.5). The overall success rate was 92.9% for caries-associated pulpitis, and 85.2% for severe tooth wear teeth, with no statistical difference (P ≥ 0.5). The treatment outcome of pulpotomy was not related to sex, tooth position, or the cause of pulpitis. The severely worn teeth, with or without secondary caries (Figs. 2, 3), have achieved the same treatment results as carious teeth.

**Table 3:** Outcomes of pulpotomy according to different characteristics of the study participants.

Variable	No. of patients/lost to follow-up	Success/failure	Success rate (%)	*p* value^a^
**Age group (years)**				
1: 18–39	47/4	43/0	100	1 vs 2: 0.043
2: 40–59	28/0	24/4	85.7	1 vs 3: 0.014
3: 60–82	30/3	22/5	81.5	2 vs 3: 0.952
**Sex**				
Male	53/1	47/5	90.4	1.0
Female	52/6	42/4	91.3	
**Tooth position**				
Maxilla	29/5	21/3	87.5	0.81
Mandibular	76/2	68/6	91.9	
**Case of pulpitis**				
Carious	75/4	66/5	92.9	0.42
Severe tooth wear	30/3	23/4	85.2	
**Total**	105/7	89/9	90.8	–

^a^Fisher’s exact test was used to determine associations between categorical variables.

**Figure 4:**
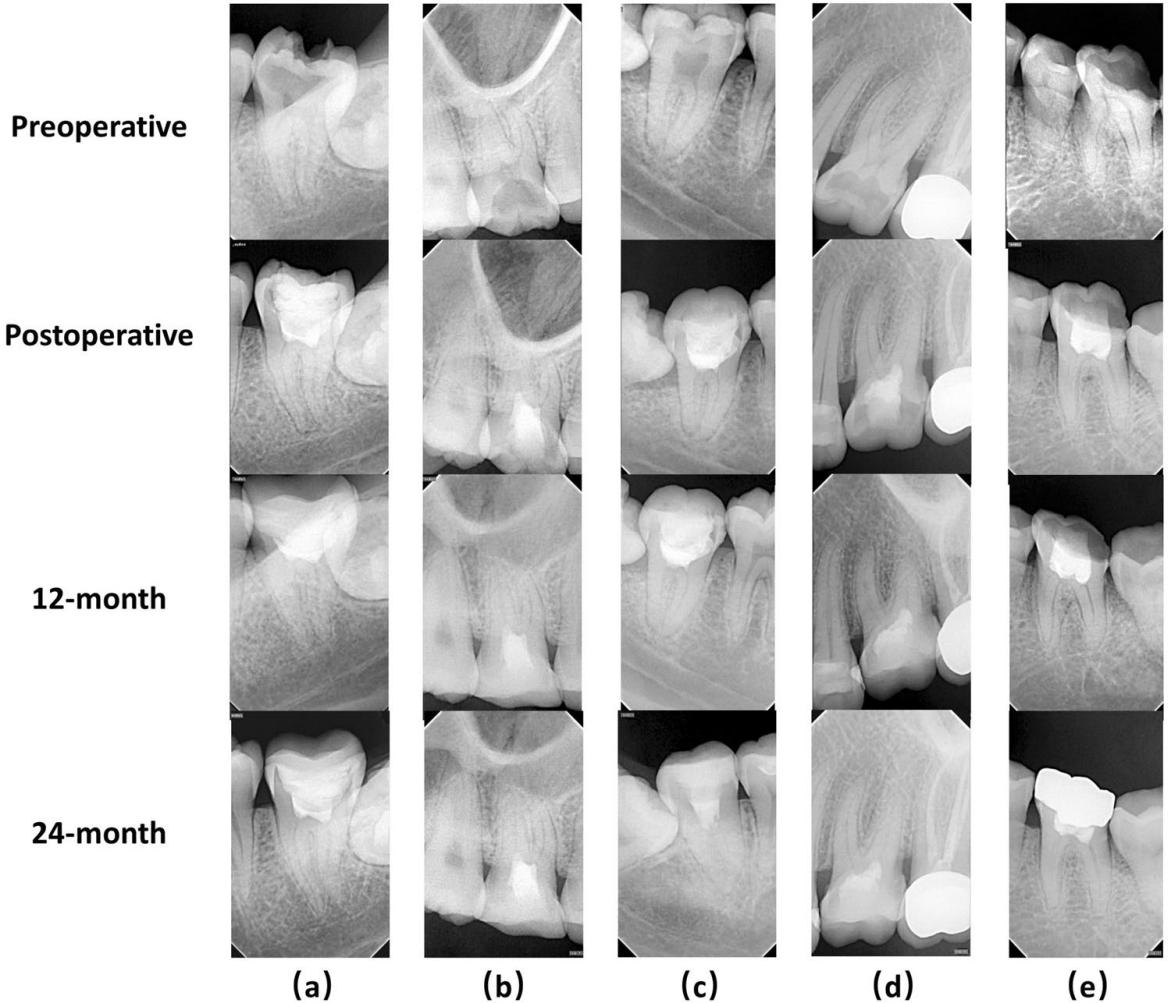
Radiographs of five pulpitis teeth included in this study. Radiographs that taken before, and immediately after the pulpotomy, at 12-month and 24-month follow-ups were shown. No obvious root canal calcification was found during the review compared to the preoperative radiographs. (**a**) Radiographs of a left mandibular second molar. (**b**) Radiographs of a right maxilla first molar. (**c**) Radiographs of a right mandibular second molar. (**d**) Radiographs of a left maxilla first molar. (**e**) Radiographs of a left mandibular first molar.

**Figure 5:**
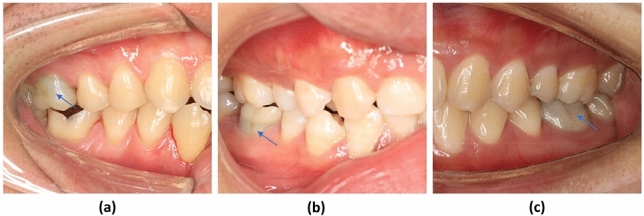
Tooth discoloration was observed at the 12-month follow-up on the teeth choosing different restoration methods after pulpotomy. (**a**) A right maxilla first molar with severe tooth wear was repaired by an inlay crown three months after pulpotomy, observed discolored at 12-month recall. (**b**) A right mandibular first molar with carious exposure was repaired by an inlay crown three months after pulpotomy and observed discolored at 12-month recall. (**c**) A left mandibular first molar with carious exposure, with a postoperative resin restoration, was observed discolored at 12-month recall.

No obvious root canal calcification was found during the review (Fig. 4). Mineralized dentin bridges appeared to be present in the root canal orifices of 15 teeth. Because the pulp cavity was not re-opened, further details were unavailable. Discoloration of the treated teeth was prevalent at the 12-month visits (Fig. 5). The study did not include anterior teeth because of esthetic considerations. All teeth were reviewed at the 12-month follow-up, and 80 teeth reached the 24-month recall. No new failed cases were found at the completed 24-month follow-up visit, and the longest-lasting case has reached 42 months.

**Figure 6:**
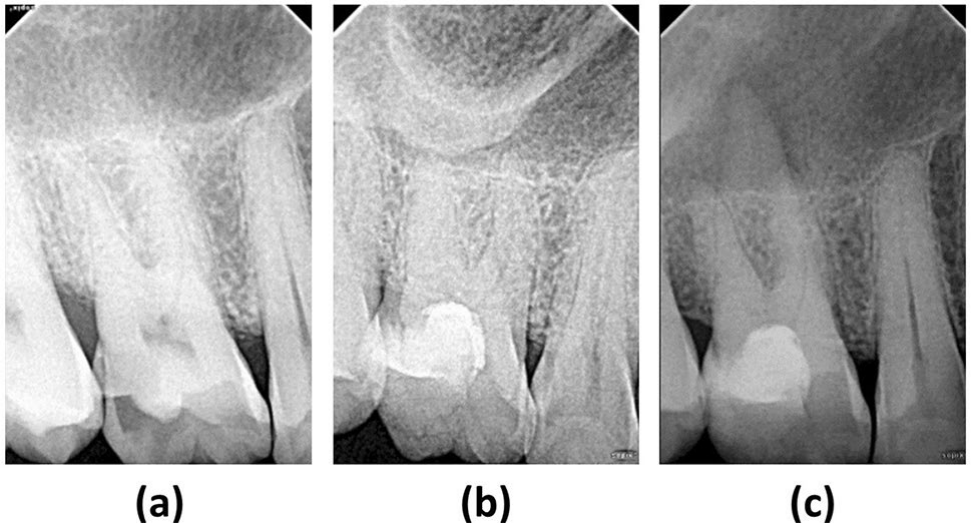
The right maxillary first molar was diagnosed with irreversible pulpitis and underwent a full pulpotomy. (**a**) Pre-operative radiographic examination showing deep caries invading the pulp chamber, with a periapical index (PAI) score of 1. (**b**) 3-month follow-up showing no abnormality. (**c**) Periapical rarefaction was observed at the 6-month follow-up, with a PAI score of 2 in the buccal roots and 3 in the palatal root.

Nine pulpotomy teeth failed because of pain, swelling, or asymptomatic periapical inflammation, for which root canal treatment was performed. Four of them failed at 2 weeks, two failed at 2 months, one at 6 months, and 2 at 12 months. The radiographs of the failure case diagnosed with apical periodontitis at the 6-month follow-up shows below (Fig. 6).

## Discussion

Compared with endodontic therapy, partial or complete pulpotomy conform to the principle of minimal invasion by maximizing the preservation of the living pulp tissue to retain its strong sensory, defense, nutrition, and regeneration functions^24^. A pulpotomy may substantially decrease the loss of tooth structure and the induced stresses caused by endodontic procedures, which would make the post-endodontically treated tooth more vulnerable to irreparable fracture^25^. Without the need for complex endodontic procedures, a pulpotomy is a more time-efficient and operator-friendly treatment. Iatrogenic endodontic complications can also be decreased. Thus, pulpotomy has been recently introduced as an option for the management of permanent teeth with irreversible pulpitis^11–15^. However, it is difficult to determine which cases are suitable for pulpotomy instead of endodontic treatment, and the factors affecting the clinical outcomes remain unclear.

In this study, 105 teeth with pulpitis caused by caries and severe tooth wear were treated by pulpotomy. The overall success rate was 90.8% at the 12-month review, demonstrating the feasibility of pulpotomy for the treatment of mature permanent teeth with symptomatic pulpitis. No statistically significant difference was found between cases with caries and those with severe tooth wear. However, age appears to have a significant impact on treatment outcomes. The success rates in group 1 (18–39 years), group 2 (40–59 years), and group 3 (≥ 60 years) were 100%, 85.7%, and 81.5% respectively, and Significant differences in success rate were found between groups 1 and 2 (P = 0.043), and groups 1 and 3 (P = 0.014). Though the small sample size of group 2 might make the statistic look significant between groups 1 and 2, the obvious statistical difference between groups 1 and 3 can also suggest that strict case selection should be considered for vital pulp therapy in older patients. It is critical to involve more cases in this study for assessing the significance of age.

The current treatment approaches for tooth hypersensitivity caused by severe tooth wear, such as topical application of desensitizing agents, adhesive applications, and laser irradiation, are often ineffective, or provide only short-term efficacy^26–28^. In some cases of severe tooth wear with persisting hypersensitivity, root canal treatment is ultimately required^27^. This study demonstrated that cases of severe tooth wear treated with pulpotomy were completely free of hypersensitivity after the treatment, suggesting that pulpotomy could be a unique solution to eliminate these symptoms. Severely worn teeth often have a narrow pulp chamber and a calcified root canal due to reparative dentin deposition in response to chronic stimuli resulting from dentin exposure^29^, making root canal treatment more difficult. In this prospective cohort study, the clinical success rate of pulpotomy in cases of severe tooth wear reached 85.2%, with no radiographic abnormalities (Figs. 2, 3) such as canal obliteration or periapical rarefaction, making it comparable to conventional root canal treatment^30^. Although the evidence gleaned from a 12-month review is insufficient, it provides preliminary proof of the practicability of treating severe tooth wear with pulpotomy. A further long-term randomized clinical trial comparing root canal treatment and pulpotomy in teeth with severe attrition and hypersensitivity should be conducted to provide more convincing evidence.

Both caries and tooth wear with pulpitis were included in this study. Vitality testing, periapical radiography, and more critically, clinical observation of the pulp tissue conditions under the microscope, were applied to confirm the vital status of the pulp tissue. Although the causes of caries and severe tooth wear are different, both can cause pulp tissue inflammatory response and subsequent infection and necrosis. Histological studies have shown that in the early stages of pulpitis, bacteria invade only the superficial layer of the pulp and then colonize the necrotic pulp. Once the pulp is infected by bacteria, it is considered irreversible pulpitis, which is incapable of self-healing^22^. However, the pulp tissue is equipped with innate and adaptive immune defense mechanisms that can confine the necrotic tissue and bacterial colonies by temporarily surrounding them with immune-inflammatory cells^22,31^. If no treatment is rendered, the pulp infection will gradually spread to the entire pulp, causing complete necrosis of the pulp tissue. In theory, if the infected pulp is promptly removed, the rest of the pulp may remain healthy; this is the basis for successful pulpotomy treatment^31^. Therefore, precisely discerning the affected pulp tissue, and removing the infected portion, could greatly improve the success rate of pulpotomy.

This study reported the outcomes of pulpotomy on permanent teeth among different aged populations: group 1 (aged 18–39 years), group 2 (aged 40–59 years), and group 3 (aged above 60 years). Age was found to be a critical factor that affected the treatment outcomes for pulpotomy. Group 1 (18–39 years) had a significantly higher success rate than groups 2 and 3 (aged above 40 years). The results corroborate a recent study that demonstrated a significant difference in the success rate of direct pulp capping between adult patients aged under 40 years and those aged over 40 years^19^. Although some of the available studies deny the effect of age on the efficacy of pulpotomy^16^, others found that deciduous teeth and young permanent teeth have a very high success rate, suggesting that age may affect the outcome of pulpotomy in mature permanent teeth^32,33^. In this study, the nine failed cases all occurred in patients aged over 40 years. The correlation between age and success rate could be attributed to the compromised reparative potential of aged pulp and its vulnerability to external insults^9^, resulting from the decreased proliferation and differentiation capacity of dental pulp stem cells, reduced root canal space, and reduced vasculature with aging^34^. Interestingly, all the failed cases occurred within 12 months, and seven out of the nine failed cases occurred within 6 months. Elmsmari et al.^35^ conducted a systematic review and concluded that partial pulpotomy is an adequate treatment option for permanent posterior teeth with carious exposures, and that 6 months can be considered a suitable period for evaluating success after a partial pulpotomy. Our finding that the majority of failed cases occurred within 6 months postoperatively supports the contention that 6 months is a critical time point for reviewing, although a long-term clinical and radiographic review at around 4 years postoperatively is essential as it is recommended for assessment of the success of root canal treatment^30,36^.

Of the nine failed cases in patients aged over 40 years, one was diagnosed with apical periodontitis 6 months postoperatively (Fig. 6). Clinical examination found that the tooth had occlusal trauma, which suggested that in addition to the factor of age, the patient’s occlusal habits and trauma history during the follow-up period may have affected the treatment outcomes. Six early failure cases presented with pain on hot and cold stimulation, a certain degree of spontaneous pain, and nocturnal pain. In four cases the pain started at 2 weeks, and in two cases, at 2 months. As previously reported, errors in preoperative diagnosis and assessment of the pulp status are likely contributing factors^37^. Another failed case presented with asymptomatic periapical periodontitis at a 1-year review, with no history of postoperative discomfort. One failed case with symptoms of acute pulpitis at the 12-month follow-up was caused by an operator error during the replacement of the crown restoration. However, long-term follow-up is still in progress, and the outcomes need to be further verified.

Some limitations should be acknowledged in this study. First, the inclusion criteria in this clinical study were comparatively conservative. According to Wolters ‘Endolight’ minimally invasive endodontic approach^38^, severe pulpitis with exacerbated pain at night is an indication for pulpotomy; however, this condition was excluded from our study. This could partially explain our relatively high success rate. Second, with MTA as the capping material, tooth discoloration commonly occurs, although this was explained to the patient before the procedure. As it showed in Fig. 5, discoloration was obviously on the treated teeth during the 12-month visits. Studies have shown that the selection of pulp capping material plays a crucial role in the preservation of vital pulp. Commonly used pulp capping materials include calcium hydroxide, bioceramic MTA, and new nano bioceramic materials. MTA has been reported to be superior to calcium hydroxide as a pulpotomy medicament^39^ because of its better biocompatibility, antibacterial properties, and edge sealing. MTA is difficult to handle, and has a long setting time, toxic elements in its composition, and the potential to cause tooth discoloration^40^. Improved materials, such as Biodentine, iRoot BP, and NeoMTA, have been developed to overcome the drawbacks and have been applied in vital pulp therapy in mature permanent teeth^41,42^. Additionally, this study only judged from a clinical point of view that aging is an important factor affecting the efficacy of pulpotomy. Further basic research at the cellular level needs to be conducted to confirm this conclusion. Finally, the follow-up time in this study was not sufficient, although further follow-ups are ongoing to closely monitor the progress of the treated teeth.

## Conclusion

Mature permanent teeth with reversible and irreversible pulpitis caused by severe tooth wear and caries were treated with pulpotomy, using MTA as a capping material, with a clinical success rate of over 90% at the 12-month review. Age was found to be an important contributing factor affecting clinical success. No statistically significant difference in clinical success was found between cases with caries and those with severe tooth wear.

## Data Availability

The data underlying this article will be shared upon reasonable request to the corresponding authors.
